# The Relation of Q Angle and Anthropometric Measures with Ankle Sprain; a Case-control study

**Published:** 2017-01-08

**Authors:** Hamid Zamani Moghadam, Seyed Taha Hoseini, Amir Masoud Hashemian, Mohammad Davood Sharifi

**Affiliations:** Department of emergency medicine, Faculty of Medicine, Mashhad University of Medical Sciences, Mashhad, Iran.

**Keywords:** Ankle Injuries, Ankle Joint, Lateral Ligament, Ankle, Emergency service, hospital

## Abstract

**Introduction:**

Since most studies on ankle sprain are medical and sports-related and not much epidemiologic and etiologic data from the general population exist in this field, the present study evaluates the relationship between Q angle and anthropometric measures with ankle sprain in the general population.

**Methods:**

In the present case-control study, all of the patients over 18 years age presenting to emergency departments (ED) of two educational Hospitals, complaining from ankle sprain, were evaluated during more than 1 year. A checklist consisting of demographic data, height, weight, body mass index (BMI), and history of ankle sprain, as well as degree of Q angle was filled for all participants. The correlation of mentioned variables with incidence of ankle sprain was calculated using SPSS 22.

**Results:**

300 patients with ankle sprain were evaluated (53.5% male). Mean age of the patients was 37.03 ± 14.20 years. Mean weight, height, and BMI were 71.71 ± 11.26 (43 – 114), 168.74 ± 8.63 (143 – 190) and 25.14 ± 3.19 (18.41 – 38.95), respectively. Mean Q angle of the patients was 12.78 ± 3.19 degrees (5 – 23). There was a significant correlation between weight (p < 0.001), BMI (p = 0.001), history of sprain (r: 0.26, p < 0.001) and Q angle (p = 0.002) with incidence of ankle sprain. In addition, there was a significant statistical correlation between weight (p = 0.031), BMI (p = 0.020) and Q angle (p = 0.004) with history of ankle sprain. In patients with a history of ankle sprain, Q angle was wider by about 2 degrees.

**Conclusion:**

It seems that the prevalence of ankle sprain directly correlates with high weight, BMI, and Q angle and is more prevalent in those with a history of sprain. Although the findings of the present study show a statistically significant correlation between these factors and ankle sprain, the correlation is not clinically significant.

## Introduction

Ankle sprain is a problem caused by injury of ligaments around the ankle and has a prevalence between 5.2 – 6 cases in 1000 population. Based on gender this rate may vary between 12.7 in 1000 for women and 0.8 in 1000 for men (1, 2). Plain radiography, magnetic resonance imaging (MRI), and clinical decision rules such as Ottawa ankle rule are among the diagnostic tools that aid in making a diagnosis in this regard. Asymmetric stretch of ankle flexor muscles, increase in body mass index (BMI), weight increase, and younger age are introduced as risk factors of ankle sprain (3, 4).

Many studies have mentioned Q angle or quadriceps angle as an independent risk factor in increasing the probability of ankle sprain (4, 5). Pefanis et al. studied 45 professional athletes and showed that there is no direct correlation between this angle and increased risk of ankle sprain (4). On the other hand, in another study the results showed the contrary and a significant correlation was found between Q angle and ankle sprain in women who played in the university basketball league in America (5). Since most studies on ankle sprain are medical and sports-related and not much epidemiologic and etiologic data from the general population exist in this field, the present study evaluates the relationship between Q angle and anthropometric measures with ankle sprain in the general population. 

## Methods

***Study design***

In the present case-control study, all of the patients presenting to the emergency departments (ED) of Imam Reza and Shahid Hasheminezhad Hospitals, Mashhad, Iran, complaining from ankle sprain, were evaluated during more than 1 year (June 2014 to October 2015). The study aimed to evaluate the correlation of Q angle and anthropometric measures with ankle sprain incidence. Written consent was obtained from the patients before including them in the study. Protocol of the study was approved by the Ethics Committee of Mashhad University of Medical Sciences. To protect personal and confidential data of patient files, the researchers adhered to the principles of Helsinki Declaration.

***Participants:***

All the patients over 18 years of age presenting to ED with complaint of ankle sprain were included using census sampling. Cases with history of trauma, fracture, and defect or disability in lower extremities were excluded. In addition, patients who had obvious open or closed fracture according to plain radiography and initial physical examination results were excluded. For all patients, a checklist consisting of demographic data, such as age, sex, height, weight, calculation of body mass index (BMI: weight/height^2^) and history of ankle sprain, was filled. Then Q angle was calculated and recorded for all the participants. An equal number of patients over 18 years old who presented to the hospital due to reasons other than trauma and lower extremity injury were included as the control group if they gave their consent.

***Q angle measurement***

**Figure 1:**
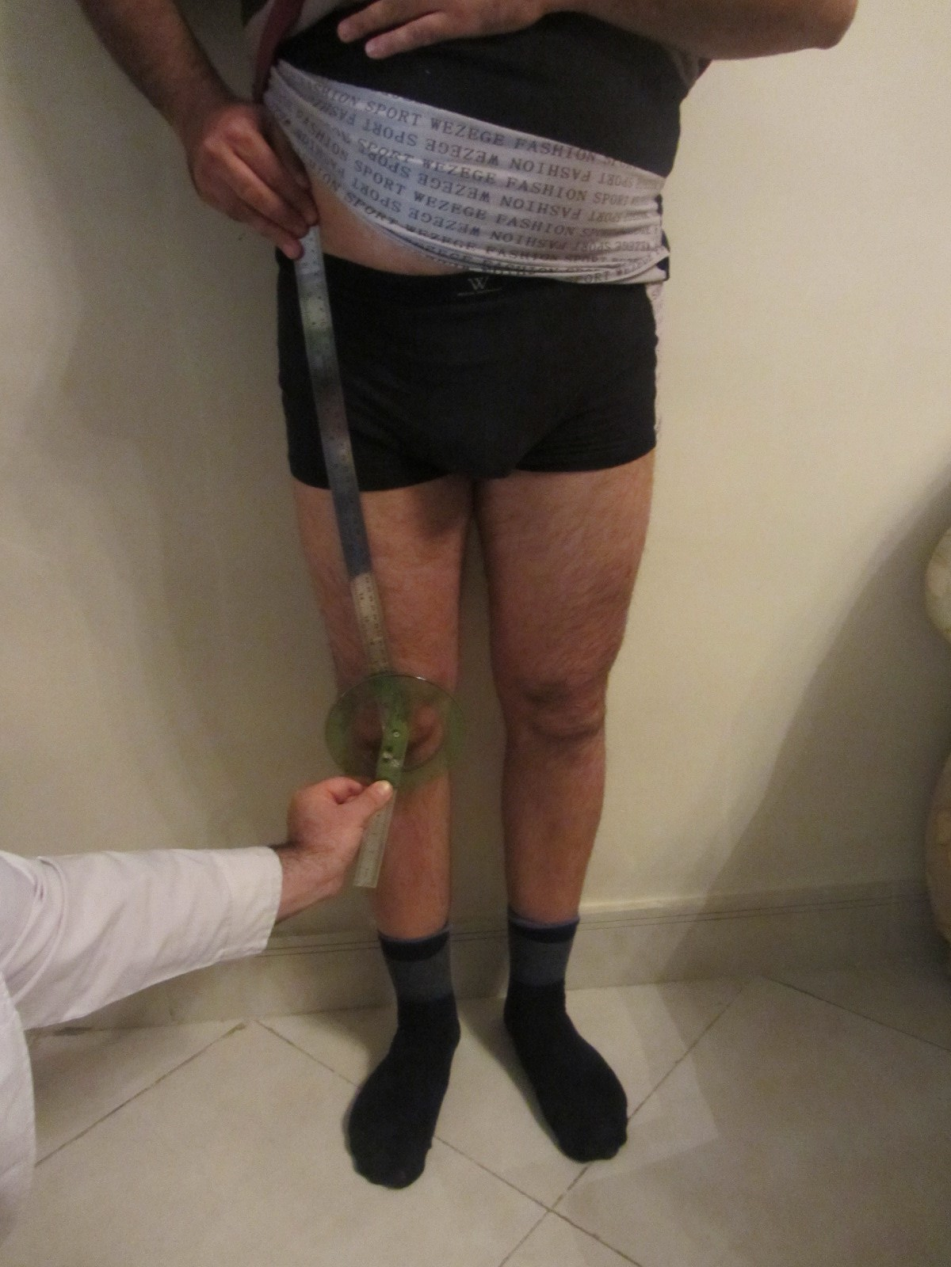
Romberg method of measuring Q angle

To calculate Q angle, anatomic points of anterior superior iliac spine, middle patellar point, tibial tuberosity while standing, without shoes and in the right lower extremity were used. The angle between the line that connects anterior superior iliac spine to middle patellar point and the line that connects tibial tuberosity to middle patellar point was considered as Q angle and was measured twice to increase accuracy. Standard universal goniometer (with 0.1 degree accuracy made by LTD Company, Japan) was used for measuring the angle. The center of the goniometer was positioned on the center of patella, its long arm parallel to or on the lateral mid line of pelvis, and the short arm on the tibial tubercle, the resulting angle was recorded. Mean of the 2 Q angle measurements were used for each patient. All the steps were done by a trained senior emergency medicine resident. Figure 1 shows Q angle measurement using Romberg method.

***Statistical analysis:***

Census sampling was used in the present study and therefore all the patients with complaint of ankle sprain who visited the ED of the mentioned hospitals during the study period were included. Statistical analysis of the data was done using SPSS version 22. Mean and standard deviation (SD) were used to report the quantitative data and frequency and percentage were used for reporting qualitative ones. After making sure that the data had normal distribution, chi-squared and t-test were applied to compare the case and control groups. Correlations were calculated using Pearson's R for categorical variables and Spearman correlation for continuous ones. P value < 0.05 was considered significant.

**Table 1:** Comparison of the case and control groups characteristics

**Variable **	**Case group**	**Control group**	**P value**
**Age (years)**	37.02 ± 14.16	37.03 ± 14.26	0.993
**Weight (kg)**	73.32 ± 11.83	70.09 ± 0.43	< 0.001
**Height (cm)**	169.20 ± 8.38	168.28 ± 8.86	0.189
**Body mass index**	25.56 ± 3.39	24.72 ± 2.93	0.001
**Q angle**	13.18 ± 3.77	12.37 ± 2.41	0.002
**Sex **			
Male	160 (53.3)	161 (53.7)	0.5
Female	140 (46.7)	139 (46.2)	
**History of sprain**	42 (14)	1 (0.3)	< 0.001

Data are shown as mean and standard deviation or frequency and percentage.

**Table 2:** Comparison of weight, height, body mass index, Q angle and gender with history of ankle sprain

**Variables **	**History of sprain**	**No history of sprain**	P value
**Age (years)**	35.63 ± 13.00	37.13 ± 14.29	0.185
**Weight (kg)**	75.28 ± 12.76	71.43 ± 11.01	0.031
**Height (cm)**	169.14 ± 9.13	168.71 ± 8.60	0.753
**Body mass index**	26.24 ± 3.30	25.03 ± 3.17	0.020
**Q angle**	14.14 ± 3.30	12.67 ± 3.13	0.004
**Sex **			
Male	22 (6.9)	299 (93.1)	0.435
Female	21 (7.5)	258 (92.5)	

Data are shown as mean and standard deviation or frequency and percentage.

## Results

300 patients with ankle sprain were evaluated (53.5% male). Mean age of the patients was 37.03 ± 14.20 years (range: 18 – 82) with the highest frequency in the 26 – 30 age group. Mean weight, height, and BMI were 71.71 ± 11.26 (43 – 114), 168.74 ± 8.63 (143 – 190) and 25.14 ± 3.19 (18.41 – 38.95), respectively. Mean Q angle of the patients was 12.78 ± 3.19 degrees (5 – 23). There was a significant correlation between weight (r: 0.127; p < 0.001), BMI (r: 0.130; p = 0.001), history of sprain (r: 0.265; p < 0.001) and Q angle (r: 0.091; p = 0.002) with incidence of ankle sprain (table 1). In addition, there was a significant correlation between weight (r: 0.084; p = 0.031), BMI (r: 0.113; p = 0.020) and Q angle (r: 0.107; p = 0.004) with history of ankle sprain. In patients with a history of ankle sprain, Q angle was wider by about 2 degrees (table 2). 

## Discussion

Based on the findings of this study, it seems that ankle sprain is more prevalent in heavy people with higher BMI and Q angle as well as those with a history of ankle sprain. There was a significant statistical correlation between weight, BMI, and Q angle with ankle sprain. However, this significant statistical correlation was not clinically significant.

Ankle sprain incidence has been estimated to be 2 in 1000 cases with significant difference between genders. In ages between 15 – 24 years the incidence is higher in males (1.62 vs 1.41), while the rate is higher in females (2.3 vs 1.65) at the age of 30. 

About 50% of ankle sprain cases have been reported while doing sports with 41% in basketball players and 9.3% in football players (6). In a study on female basketball players in America, a significant correlation was found between Q angle and ankle sprain, which is in line with the findings of the present study (5). In another study, a significant correlation was found between the anatomic status of ankle joint and prevalence of sprain (7). In addition, in professional football players asymmetric stretch of ankle, BMI, and gaining weight were found to be affecting ankle sprain incidence. Old age and asymmetric stretch of ankle ligaments have been factors resulting in poor improvement. However, young people are more at risk of ankle sprain (3). Hagglund et al. showed that despite the history of hamstring and knee injuries increasing the risk of being injured again by 3 to 4 times, history of ankle sprain does not increase the risk of it happening again (8). Generalized joint laxity, ankle ligament fixation, and ankle stretch rate were highly related to ankle sprain incidence (9). In general, factors that cause an unbalance in pressure on the joints of lower extremities lead to injuries such as sprain and stretch in the joints of this region. Q angle is responsible for transmitting pressure from pelvis to legs and has been estimated to be about 10 degrees in men and 15 degrees in women. If this angle deviates from its normal position, in addition to causing disorders in patellofemoral function (including patellofemoral pain, and patellar imbalance), it may be a risk factor for ankle injuries. By using preventive measures to avoid ankle stretch (such as wearing shoes with ankle support) we may be able to avoid sprain. Activity level of the participants, their occupation, race, and factors affecting muscular strength or tendon and joint stability were among the confounding factors of this study that should be evaluated in future studies along with other effective factors in ankle, knee and pelvis regions.

**Limitations:** Small sample size and not considering all probable risk factors of ankle sprain were among the most important limitations of the present study.

## Conclusion:

It seems that the prevalence of ankle sprain directly correlates with high weight, BMI, and Q angle and is more prevalent in those with a history of sprain. Although the findings of the present study show a statistically significant correlation between these factors and ankle sprain, the correlation is not clinically significant.
